# Clinical use of antimicrobial regional limb perfusion in adult horses diagnosed with synovial sepsis or penetrating synovial wounds at a single equine referral hospital in the Midwest United States—163 cases (2010–2020)

**DOI:** 10.3389/fvets.2025.1504486

**Published:** 2025-03-26

**Authors:** Zach Loppnow, Kevin Kersh, Chong Wang, Sienna Spaethe, Jarrod Troy

**Affiliations:** ^1^Steinbeck Peninsula Equine Clinics, Salinas, CA, United States; ^2^Iowa State University College of Veterinary Medicine, Veterinary Clinical Sciences, Ames, IA, United States; ^3^Department of Veterinary Diagnostic and Production Animal Medicine, Iowa State University College of Veterinary Medicine, Ames, IA, United States; ^4^Department of Statistics, Iowa State University, Ames, IA, United States

**Keywords:** equine, intravenous regional limb perfusion, synovial, sepsis, penetrating wound

## Abstract

The clinical outcomes in horses diagnosed with established synovial sepsis (SS) or minimally contaminated synovial wounds (SW)/non-established sepsis, following intravenous regional limb perfusion (IVRLP) treatment, have not been reported since 2010. Additionally, previous reports on this subject were confined to just two clinical retrospective reports. This study aimed to provide an update on the short-term (hospital discharge) and long-term (≥1 year) survival rates in adult horses diagnosed with SS or SW treated with IVRLP at a single institution in the USA from 2010–2020. The study also seeks to determine IVRLP variables associated with survival. The medical records of 163 adult equine, either diagnosed with SS (group 1) or SW (group 2), from 2010–2020 treated with IVRLP were reviewed. The short-term survival rate was 88.9% (56 out of 63) for group 1 and 99.0% (99 out of 100) for group 2. The long-term survival rate was 65.1% (41 out of 63) for group 1 and 83.6% (46 out of 55) for group 2. Gentamicin was the most commonly chosen IVRLP antimicrobial choice (153 out of 163 cases). Horses in group 1 were less likely to survive until hospital discharge (*p* = 0.01; odds ratio [OR] = 0.08; 95% confidence interval [CI]: [0.01, 0.68]). The total number of IVRLPs performed during hospitalization was significantly associated with non-survival to hospital discharge (*p* = 0.01; OR = 0.47; 95% CI: [0.22, 0.87]), indicating that horses were less likely to survive as the total number of IVRLPs increased. No variables were associated with long-term survival. Overall survival in adult horses diagnosed with SS or SW after treatment is good, and this study provides an updated clinical report on adult equine SS or SW cases that received IVRLP in the USA. This study also reports IVRLP variables, including the total number of IVRLPs and the number of consecutive IVRLPs, that may be associated with short-term survival.

## 1 Introduction

Septic synovitis is a common, life-threatening condition in adult equids that can lead to lifelong lameness despite treatment (1–5). Septic synovitis (SS) often develops after bacterial invasion through penetrating synovial wounds, hematogenous spread, or iatrogenic contamination (1–5). The consequences of developing SS are so severe in that even minimally contaminated synovial wounds or lame horses with synovial effusion, which may indicate synovial contamination (SW), are treated as septic until proven otherwise (1–5). The treatment of SS and SW typically involves generating high intrasynovial antimicrobial concentrations and/or synovial lavage (1–5). Antimicrobial intravenous regional limb perfusion (IVRLP) constitutes a therapeutic technique that produces elevated intrasynovial antimicrobial concentrations within the distal limbs of equine. This method is routinely employed by equine practitioners for treatment of SS and SW (1–5). Although numerous studies have evaluated experimental IVRLP use and/or administration techniques in horses, only three reports focus on clinical IVRLP application and survival outcomes in horses with SS or SW (1–11). Two reports describe clinical equine IVRLP administration, including associated complications and outcome in horses diagnosed with SS, lacerations (intrasynovial or extrasynovial), and other miscellaneous conditions (1, 2). Although these reports provide valuable insights, they exclusively document cases that were submitted in 2010 and originated from equine referral institutions located outside the United States (1, 2). The third report originated from a referral institution within the United States and focused exclusively on comparing only gentamicin and meropenem IVRLP and SS survival with IVRLP treatment (3). This report fails to address other clinical aspects of equine IVRLP or SW outcomes (3). Investigating the clinical effects of IVRLP treatment for equine SS and SW is challenging. Therapeutic recommendations are often guided by a combination of both experimental and clinical investigations (1–11). Evaluating the clinical use of equine IVRLP across different geographic locations is also important, as common bacterial isolates cultured from equine SS cases have been shown to vary by geographic location (12–15). Furthermore, antimicrobial-resistant bacterial strains continue to develop over time, making it important to provide ongoing clinical updates regarding the developments (15). This study aimed to update on the short-term (survival to hospital discharge) and long-term (≥1 year) survival rates for adult horses diagnosed with SS or SW between 2010 and 2020 at a single institution in the Midwest United States treated with IVRLP and identify IVRLP variables associated with survival. The hypothesis was that none of the variables associated with IVRLP administration—such as number of IVRLPs performed, administration intervals, antibiotic type, tourniquet times, perfusate volumes, or the inclusion of local anesthetic—would significantly affect short- or long-term patient outcomes.

## 2 Methods

### 2.1 Case selection and definition of clinical groups

Medical records of horses diagnosed with a septic synovial structure or suspected synovial contamination that received IVRLP antimicrobial treatment while hospitalized at the Iowa State University Lloyd Veterinary Medical Center (ISU LVMC) from January 2010 to December 2020 were reviewed. A case was defined as a single hospitalization event for an individual horse. If a horse was discharged alive from the hospital and presented back for treatment, it was considered as a new, separate case. If a horse diagnosed with septic synovitis or synovial wound did not receive IVRLP, then they were not included in the study. Records were excluded if the horse was below 12 months of age.

Horses were included in the study if they had a synovial wound or laceration, or if they were lame due to synovial effusion following synovial endoscopy, synovial injection, or other causes that could allow bacterial penetration into a synovial structure. Horses were categorized into two groups based on the reported diagnostics, which included joint distension, fluid analysis, cytology, and/or bacterial culture. Group 1 was identified as having established synovial sepsis, defined as any instance with bacterial visualization on synovial fluid cytology, a positive bacterial culture from synovial fluid, or synovial fluid analysis that satisfied at least three of the following conditions: WBC ≥ 30,000 cell/μL (normal range: 50–500 cells/μL), >90% polymorphonuclear leukocytes (normal range: <10%), total protein >4 g/dL (normal range: 0.8–2.5 g/dL), presence of fibrin in the synovial space, and/or evidence of degenerative changes in polymorphonuclear leukocytes (1, 16). Group 2 was classified as suspected synovial contamination, encompassing any case with fewer than two of the synovial fluid analysis conditions listed for Group 1 or cases in which the synovial structure was affected but may not have had fluid analysis documented in the records. The cases were categorized in this manner to compare IVRLP variables between those with established synovial sepsis and without, as well as the outcomes following IVRLP treatment. These criteria were aimed to minimize subjective evaluation of established septic synovitis vs. minimal contamination or inflammation when comparing cases and IVRLP variables.

### 2.2 Variables obtained from review of medical records

#### 2.2.1 Demographic information

Collected data included signalment (breed, sex, and age) as well as the athletic discipline or purpose of use.

#### 2.2.2 Preadmission information

The data collected regarding prehospital admission included the presenting complaint, the duration of clinical symptoms prior to hospital admission, and the affected limb(s).

#### 2.2.3 Hospital diagnostics

Information collected at admission and during hospitalization included a subjective lameness evaluation, the type of synovial structure involved, synovial fluid analysis and cytology, as well as bacterial culture of the synovial fluid.

#### 2.2.4 IVRLP treatment variables

The variables recorded related to IVLRP technique included the time after admission when IVRLP treatment was initiated, the total number of IVRLP sessions during hospitalization, the number of IVRLP treatments given on consecutive days (if the treatment was stopped and restarted during the same hospitalization), the time interval between IVRLP administrations, and the vein used for IVRLP. The tourniquet variables recorded included the type of tourniquet, the number of tourniquets used on a single limb, and the duration until the tourniquet removal. The recorded variables for the perfusate solution included total IVRLP perfusate volume and if local anesthetic was combined with the IVRLP perfusate. Time interval (or “break”) between IVRLP treatments was defined as the duration from the administration of the last reported IVRLP until the next reported IVRLP. If no additional IVRLP was reported, then no time “break” was noted. In this study, a “break” was included, as it is typical practice at this institution to perform IVRLP every 24 h for 3 consecutive days, followed by a 24-h break without IVRLP before resuming it for 3 consecutive days. These 3-day IVRLP intervals followed by a “break” were performed to reduce the risk of phlebitis in the IVRLP target vein, allowing for its future use, even if no clinical signs of phlebitis were apparent.

Additional recorded variables included whether IVRLP was performed with standing sedation or under general anesthesia (GA), the antimicrobial type used, the antimicrobial dose, and any changes in antimicrobial type during hospitalization. General anesthesia was defined as cases anesthetized and maintained under anesthesia using total intravenous anesthesia and/or inhalant.

#### 2.2.5 Other treatment variables

The majority of the horses received treatments other than IVRLP while hospitalized. The data were recorded if surgical lavage of the synovial structure was performed, including the type of lavage performed (needle-through-needle [NTN] or arthroscopic), the number of lavages performed, and the intervals between them. Antimicrobial use was noted for both the routes (systemic and intrasynovial routes of administration) and type in each case.

#### 2.2.6 Outcome variables

Prior to death or discharge, a lameness assessment was performed, and any changes in lameness following treatment were recorded. The duration of hospitalization was also noted. Both variables were examined as outcomes in statistical modeling.

Short-term outcomes were defined as survival to hospital discharge or euthanasia/death prior to hospital discharge based on medical records. The reasons for euthanasia/death prior to hospital discharge were obtained from the medical records. The long-term outcome was assessed at the time of follow-up, which took place more than 1 year after hospital discharge, using a standardized telephone questionnaire (Supplementary Document S1) with the owner or primary agent noted in the medical record. The follow-up questions were aimed to determine whether the horse was alive, had been euthanized due to the previously diagnosed septic synovitis or a penetrating wound, euthanized or died from an unrelated reason, demonstrated lameness in the treated limb, if additional synovial cultures had been conducted, and if the horse had returned to its previous level of work.

The global variables assessed for their impact on short-term outcome, other than IVRLP variables, included the following: The categorization into groups 1 and 2, a positive synovial fluid culture, synovial fluid cell counts, intraarticular antibiotic administration, endoscopic surgical treatment, needle-through-needle lavage, and duration of hospitalization. To account for potential influences from improvements in veterinary medical therapy over time and developments in IVRLP techniques, it was noted whether the patient was treated between 2010 and 2015 or between 2016 and 2020, and this was evaluated for its effect on short-term outcomes.

### 2.3 Data analysis

The descriptive statistics were performed for all data and for the data within each group. The data were assessed for normality by Shapiro–Wilk tests. Factors such as sex, synovial structure involved, the type of surgical intervention, the duration of hospitalization, IVRLP administration site, the initial IVRLP performed under sedation or general anesthesia, the type of antibiotic used in IVRLP, the total volume of IVRLP, the incorporation of local anesthetic into the IVRLP, the type of local anesthetic used, the application of a perineural nerve block, the number of IVRLPs performed prior to a break, the total number of IVRLPs, the time intervals between IVRLPs, the intraarticular antibiotic use, the organisms seen on cytology, the presence of fibrin in synovial space, and the results of bacterial culture were included in logistic models to assess variables associated with survival within each group. Cohort categorization, positive bacterial culture, synovial fluid cell counts, intraarticular antibiotic administration, use of endoscopic surgical treatment, use of needle-through-needle lavage surgical treatment, duration of hospitalization, and presentation between 2010 and 2015 or 2016–2020 were included in logistic models to assess variables associated with survival for the total population. A simple exact logistic regression analysis was conducted to evaluate the association between the short-term survival outcome and each explanatory variable of interest. The association between the categorical variables was assessed using a Fisher’s exact test. The variables with *p* < 0.05 were considered significant. Furthermore, multiple logistic regression analyses were also performed within each group to develop a model for the survival outcome. The backward elimination method was applied for model selection in the multiple logistic regression, using an inclusion significance level of 0.1. The odds ratios and 95% CIs were calculated and reported.

## 3 Results

### 3.1 Demographic information

Among the 163 cases of 159 equines, 63 (38.7%) met the inclusion criteria for group 1, while 100 (61.3%) were included in group 2.

The horses included in group 1 had a median age of 9 years (age range: 1–27 years) and comprised 32 (50.8%) geldings, 28 (44.4%) mares, and 3 (4.8%) stallions. The horse breeds represented in group 1 included the following: Quarter Horse: 32 (50.8%); Paint Horse: 6 (9.5%); Thoroughbred: 4 (6.3%); Appaloosa: 4 (6.3%); Standardbred: 3 (4.8%); Percheron: 3 (4.8%); Missouri Fox Trotter: 3 (4.8%); Trakehner: 2 (3.2%); Saddlebred: 2 (3.2%); and 1 (1.6%) of each of the following: National Show Horse, Hanoverian, Arabian, and mix breed. Athletic use of these horses recorded at the time of admission included 36 Western pleasure (57.1%), 7 racing (11.1%), 7 broodmares (11.1%), and 1 (1.6%) each of the following: English riding, breeding stallion, barrel racing, roping horse, show horse, pulling, riding lessons, games, and sport horse. In 4 (6.3%) cases, the athletic use was not reported.

Horses included in group 2 had a median age of 7.5 years (age range: 1–26 years) and included 48 (48%) geldings, 49 (49%) mares, and 3 (3%) stallions. Breeds represented included the following: Quarter Horse: 56 (56%); Paint: 13 (13%), Thoroughbred: 10 (10%), and 2 (2%) of each of the following breeds: Percheron, Appaloosa, Missouri Fox Trotter, Saddlebred, Belgian, Warmblood, Tennessee Walking Horse, and 1 (1%) each of the following breeds: Standardbred, Arabian, Holsteiner, Shetland, Oldenburg, Mustang, Shetland pony, and Hackney pony. Athletic use of these horses recorded at the time of admission included 49 Western pleasure (49%), 12 racing (12%), 9 barrel racing (9%), 8 broodmare (8%), 3 sport horse (3%), 2 Show Horse (2%), 2 English pleasure (2%), 2 breeding stallions (2%), 2 reining (2%), and 1 (1%) each of the following: rodeo, roping horse, and games. In 8 (8%) cases, athletic use was not reported.

### 3.2 Preadmission information

In the 63 horses of group 1, the presenting complaints were recorded. These included 30 synovial laceration or wounds (47.6%), 18 cases of septic synovitis suspected by the referring veterinarian (28.6%), 9 cases of unknown lameness (14.3%), 3 instances of lameness following synovial injection (4.8%), and 3 cases of postoperative orthopedic surgery lameness (4.8%). The median duration of clinical signs prior to hospital admission was 1 day (interquartile range [IQR]: 1–4 days) with the affected limbs as follows: 20 left front (31.7%), 17 right hind (27%), 16 left hind (25.4%), 9 right front (14.3%), and 1 with both hinds (1.6%).

Among the 100 horses of group 2, the following complaints were presented: 81 cases of synovial laceration or wounds (81%), 12 cases of septic synovitis suspected by the referring veterinarian (12%), 3 cases of unknown lameness (3%), 2 cases of lameness following synovial injection (2%), and 1 each (1%) of foot abscess communication and postoperative orthopedic surgery lameness. The median duration of clinical signs prior to hospital admission was 1 day (IQR: 0–7 days) with the following limbs affected: 35 left hind (35%), 24 right front (24%), 24 right hind (24%), 16 left front (16%), and 1 with both hinds (1%).

### 3.3 Hospital diagnostics

Among the 63 horses in group 1, the subjective lameness scores (American Association of Equine Practitioners [AAEP] Grading Scale 1–5) (17) at admission were recorded in 57 (90.4%) of the cases, indicating a median lameness grade of 4/5 (IQR: 4–5). A total of 54 (85.7%) cases had a single septic synovial structure involved with the most commonly affected structures being the digital flexor tendon sheath (29.6%), distal interphalangeal joint (22.2%), or tarsocrural joint (14.8%). In 9 (14.3%) cases, multiple septic synovial structures were identified, including 2 cases with both the distal interphalangeal joint and navicular bursa (22.2%), 2 cases with tarsocrural and distal intertarsal joints (22.2%), 2 cases with radiocarpal and intercarpal joints (22.2%), and 1 (11.1%) case each with tarsocrural joint and calcanean bursa, metacarpophalangeal joint and digital flexor tendon sheath, as well as the digital flexor tendon sheath and navicular bursa affected. The synovial fluid analysis and cytology results were available for 59 (93.7%) cases. The fluid cytology identified bacterial organisms in 12 (20.3%) of the fluid samples. These bacterial organisms included the following: Rods: 4 (33.3%); cocci: 4 (33.3%); rods and cocci: 2 (16.7%); filamentous rods: 1 (8.3%); or diplococci: 1 (8.3%). All 59 cases with fluid samples were submitted for bacterial culture, resulting in a positive bacterial culture in 47 (79.7%) cases with a median culture time of 5 days (IQR: 4–6 days). The organisms most commonly identified in the culture were *Streptococcus equi* subsp. *Zooepidemicus* (23.4%), *Escherichia coli* (17%), and *Staphylococcus aureus* (12.8%). Three of the 47 cases had multidrug-resistant organisms identified on culture: *Staphylococcus epidermicus*, *Enterobacter* sp., and methicillin-resistant *Staphylococcus aureus* (MRSA).

For the 100 horses in group 2, the admission lameness scores were recorded in 68 (68%) cases (median grade 4/5, IQR: 4–4) (17). Overall, 96 (96%) cases involved a single septic synovial structure. The digital flexor tendon sheath (26%), distal interphalangeal joint (18.8%), and the proximal interphalangeal joint (11.5%) were the most commonly affected structures. In 3 (3%) cases, multiple synovial structures were identified, including a digital flexor tendon sheath and calcaneal bursa in separate limbs (33.3%), a distal interphalangeal joint and digital flexor tendon sheath in the same limb (33.3%), and a metacarpophalangeal joint and distal interphalangeal joint in the same limb (33.3%) A single case involved three separate synovial structures, including a hind distal interphalangeal joint, hind navicular bursa, and an extensor carpi radialis tendon sheath. All cases of group 2 had initial synoviocentesis performed with synovial fluid obtained in 33 (33%) cases. All collected synovial fluid samples were sent for analysis. No organisms were detected in cytology of any of the synovial fluid samples for group 2. All samples were sent for bacterial culture, and all bacterial cultures turned negative for group 2.

### 3.4 IVRLP treatment variables

Table 1 shows case numbers and median (IQR) for investigated IVRLP treatment variables in each group.

**Table 1:** Intravenous regional limb perfusion variables for SS vs. SW (median, [range]).

	Group 1 (SS)		Group 2 (SW)	
Time to first IVRLP (h)		Number of cases		Number of cases
	0	57	0	89
	12	3	12	4
	24	2	24	5
	192	1	72	1
			120	1
Number of IVRLPs (median)	Total	Prior to break	Total	Prior to break
	4	3	4	3
Time between IVRLP (h)	12	1	12	2
	24	60	24	88
	48	2	48	6
			60	1
			90	1
			184	1
IVRLP administration sites		Number of cases		Number of cases
	Saphenous	31	Saphenous	38
	Palmar/plantar digital	18	Palmar/plantar digital	35
	Cephalic	12	Cephalic	21
	Palmar metacarpal	1	Palmar metacarpal	1
	Plantar metatarsal	1	Plantar metatarsal	4
Initial IVRLP		Cases		Cases
	Anesthetized	46	Anesthetized	70
	Standing	17	Standing	30
IVRLP antimicrobial		Cases		Cases
	Gentamicin	57	Gentamicin	96
	Ceftiofur sodium	3	Meropenem	3
	Meropenem	2	Amikacin	1
	Amikacin	1		
Tourniquet duration (min) median (IQR)	30 (1.25)		30 (5)	
perfusate volume (mL) median (IQR)	60 (0)		60 (0)	
Local anesthetic used		Number of cases		Number of cases
	2% Mepivacaine	36	2% Mepivacaine	69
	2% Lidocaine	1	2% Lidocaine	2

Among the 63 cases of group 1, IVRLP was performed in 57 (90.5%) cases upon admission, 12-h later in 3 (4.8%) cases, 24 h later in 2 (3.2%) cases, and 192 h in 1 (1.6%) case. The median total number of IVRLPs during hospitalization per horse was 4 (IQR: 3–6) with IVRLP performed a median number of 3 (IQR: 3–4) times consecutively before a break in IVRLP treatment. The time interval between consecutive IVRLP administrations was 24 h in all cases except for three cases; 1 case had a time interval of 12 h and 2 cases had intervals of 48 h. The group 1 IVRLP administration sites included 31 saphenous vein (49.2%), 18 palmar or plantar digital vein (28.6%), 12 cephalic vein (19%), 1 palmar metacarpal vein (1.6%), or 1 plantar metatarsal vein (1.6%). In the context of digital veins, the records did not specify whether the medial or lateral veins were selected for the IVRLP. It has been reported that the target vein remained consistent throughout all instances of IVRLP for a particular case. The majority of the cases in group 1 underwent initial IVRLP performed under general anesthesia (46 cases, 73.0%), while 17 (26.9%) cases were performed under standing sedation. Gentamicin was the most commonly used antimicrobial for initial IVRLP treatment in 57 (90.5%) cases, with a median amount of 1,000 mg (IQR: 1,000–1,000 mg) in each IVRLP. Additional IVRLP antimicrobials included ceftiofur sodium 3 (4.8%), meropenem 2 (3.2%), and amikacin 1 (1.6%). In the 57 cases initially receiving gentamicin IVRLP, 14 (24.6%) cases were switched to another antimicrobial (median: 7 days; IQR: 3.5–10.5 days) after starting IVRLP therapy. IVRLP antimicrobial changes were made due to bacterial culture sensitivity results or clinician discretion. Gentamicin IVRLP antimicrobial was modified to the following: Meropenem: 7 (50%) cases; amikacin: 2 (14.3%) cases; imipenem: 2 (14.3%) cases; ceftiofur sodium: 1 (7.1%) cases; ceftazidime: 1 (7.1%) case; or enrofloxacin: 1 (7.1%) case. In all cases of group 1, a wide rubber Esmarch tourniquet (10-cm width) was used for IVRLP; however, it was not recorded whether more than one tourniquet per limb was applied during IVRLP. Tourniquet duration was reported in 16 (27.0%) cases with a median duration of 30 min (IQR: 28.75–30 min). Total IVRLP perfusate volume was recorded in 29 (46%) group 1 cases with a median volume of 60 mL (IQR: 60–60 mL). The local anesthetic was combined with the IVRLP perfusate in 37 (58.7%) cases of group 1 with 36 (97.3%) of them using 2% mepivacaine and 1 (2.7%) using 2% lidocaine. The median amount of local anesthetic used was 200 mg for 2% mepivacaine and 200 mg in the 2% lidocaine case.

In the 100 cases of group 2, IVRLP was initiated at admission in 89 (89%) cases, 12 h in 4 (4%) cases, 24 h later in 5 (5%) cases, 72 h later in 1 (1%) case, and 120 h in 1 (1%) case. The median total number of IVRLPs during hospitalization per horse was 4 (IQR: 3–5) with IVRLP consecutively performed a median number of 3 (IQR: 2–3) times. In the cases of group 2, the time interval between consecutive IVRLP administrations was 24 h in 88 (88.9%) cases, 48 h in 6 (6.1%) cases, 12 h in 2 (2%) cases, and 1 case each with intervals of 60, 90, and 184 h. One case in this group received only a single IVRLP. Group 2 cases for IVRLP administration sites included the saphenous vein (38 cases, 38%), palmar or plantar digital vein (35 cases, 35%), cephalic vein (21 cases, 21%), palmar metatarsal veins (4 cases, 4%), and palmar metacarpal vein (1 case, 1%), along with 1 case with 2 separate vein sites (cephalic and saphenous). For digital veins, the records did not specify whether medial or lateral veins were selected for the IVRLP. The target vein reportedly remained unchanged throughout all IVRLP in a specific case. The majority of the cases in group 2 had the initial IVRLP performed under general anesthesia (70 cases, 70%), while 30 cases (30%) were performed under standing sedation. Gentamicin was the most commonly used antimicrobial for initial IVRLP treatment in 96 (96%) cases out of 100 cases with a median amount of 1,000 mg (IQR: 1,000–1,000 mg) in each IVRLP. Additional IVRLP antimicrobials included meropenem 3 (3%) and amikacin 1 (1%). IVRLP antimicrobials were changed in 3 cases a median of 2 days (IQR: 2–2.5 days) after starting IVRLP therapy. IVRLP antimicrobial changes were made at the clinician’s discretion. In the 3 cases that IVRLP antimicrobials changed, they were switched from amikacin to gentamicin in 2 (66.7%) cases and meropenem to amikacin in 1 (33.3%) case. In all cases of group 2, a wide rubber Esmarch tourniquet was used for IVRLP, but it was not recorded if more than 1 tourniquet per limb was used. Tourniquet duration was reported in 24 (24%) cases, with a median duration of 30 min (IQR: 25–30 min). The total IVRLP perfusate volume was recorded in 60 (60%) cases, with a median volume of 60 mL (IQR: 60–60 mL). The local anesthetic was combined with the IVRLP perfusate in 71 (71%) cases of group 2. Among those 71 cases, 69 (97.2%) cases used 2% mepivacaine and 2 (2.8%) cases used 2% lidocaine. The median amount of local anesthetic used was 200 mg for both 2% mepivacaine and 2% lidocaine.

### 3.5 Other treatment variables

In group 1, synovial lavage was recorded for 61 cases: synovial endoscopy 21 (34.4%) cases, NTN lavage 30 (49.1%) cases, or a combination of NTN lavage and synovial endoscopy 10 (16.4%). The time from admission until lavage was recorded in 31 (49%) cases out of the 61 cases with a median of 0 h (IQR: 0–12 h) after admission. Endoscopy was repeated in 2 cases, 1 case within 24 h postoperatively and 1 case 8 days postoperatively. NTN lavage was repeated in 10 cases, with 7 receiving 2 NTN lavages, 1 receiving 3 NTN lavages, 1 receiving 4 NTN lavages, and 1 receiving 5 NTN lavages. The systemic antimicrobial administration during hospitalization was recorded in 62 cases with 58 (93.5%) cases receiving two antimicrobials concurrently and 4 (6.5%) cases receiving only one antimicrobial. Gentamicin in combination with procaine penicillin G (PPG) was the most commonly administered systemic antimicrobial combination in the 62 cases, with 47 (81%) cases initiated at presentation for a median duration of 6 days (IQR: 4–8.5 days). The systemic antimicrobial therapy was changed in 36 (58%) cases out of 62 cases due to culture sensitivity results, change from injectable to oral antimicrobials prior to hospital discharge, or attending clinician discretion. In those 36 cases, antimicrobial therapy was changed to sulfamethoxazole and trimethoprim (SMZ/TMS) in 30 (83.3%) cases, chloramphenicol in 2 (5.6%) cases, metronidazole in 2 (5.6%) cases, ceftiofur crystalline free acid in 1 (2.8%) case, or enrofloxacin in 1 (2.8%) case.

Intrasynovial antimicrobial administration was reported in 22 cases of group 1 with 18 (81.8%) receiving a single intrasynovial antimicrobial injection during the initial lavage or synoviocentesis, and 4 (18.2%) receiving intrasynovial antimicrobial continuous rate infusion (CRI) in the affected area. Intrasynovial antimicrobials administered by injection included gentamicin 14 (77.8%) cases and amikacin 4 (22.2%) cases. All cases that received intrasynovial gentamicin also received IVRLP with gentamicin. The four cases that received intrasynovial amikacin received IVRLP containing gentamicin (1,000 mg; 2 cases), meropenem (1,000 mg; 1 case), or amikacin (1,000 mg; 1 case). The cases that were treated with intrasynovial antimicrobial CRI received gentamicin (3 cases, 75%) or ceftiofur sodium (1 case, 25%). The case using ceftiofur sodium for CRI also received an intrasynovial injection of gentamicin.

For group 2, synovial lavage was recorded for 92 cases: synovial endoscopy in 23 (25%) cases and NTN lavage in 69 (75%) cases. No case in group 2 received both NTN and endoscopic lavages or more than one surgical lavage during hospitalization. The time from admission until lavage was recorded in 23 (25%) cases out of the 92 cases with a median of 0 h (IQR: 0–12 h) after admission. Systemic antimicrobial administration during hospitalization was documented in 99 cases with 75 (75.8%) cases receiving two antimicrobials concurrently and 24 (24.2%) cases receiving only a single antimicrobial. Gentamicin combined with procaine penicillin G (PPG) was the most commonly administered systemic antimicrobial combination in the 99 cases, with 65 (86.7%) initiated at the time of presentation for a median duration of 5 days (IQR: 4–7 days). Systemic antimicrobial therapy was changed in 44 cases of group 2 due to a change from injectable to oral antimicrobials prior to hospital discharge or attending clinician discretion. Antimicrobial therapy was changed to SMZ/TMS in 40 (90.9%) cases, ceftiofur crystalline free acid in 3 (6.8%) cases, or enrofloxacin in 1 (2.3%) case.

Intrasynovial antimicrobial administration was reported in 23 cases in group 2, with 16 (69.6%) receiving a single intrasynovial antimicrobial injection during the initial lavage or synoviocentesis and 5 (21.7%) cases receiving intrasynovial antimicrobial CRI in the affected structure. 2 (8.7%) received both intrasynovial injection and CRI. Intrasynovial antimicrobials administered by injection included gentamicin (12 cases, 75%), amikacin (3 cases, 18.8%). All intrasynovial injection cases received gentamicin in the IVRLP, except for one amikacin case, which received a ceftiofur IVRLP. The cases that were treated with intrasynovial antimicrobial CRI received gentamicin (4 cases, 80%) or ceftiofur sodium (1 case, 20%). All intrasynovial CRI cases received gentamicin IVRLP. One case each was treated with amikacin or gentamicin as both intrasynovial injection and CRI.

### 3.6 Outcomes

Among the 63 cases of group 1, lameness scores were recorded at discharge in 46 (83.6%) cases, with at least one subjective improvement in lameness score (according to AAEP) observed within a median of 1 day after presentation (IQR: 1–4 days). Horses were hospitalized for a median of 9 days (IQR: 4–91). Short-term survival was 88.9% (56 cases) with 7 (11.1%) cases euthanized due to unresolving synovial sepsis, persistent lameness, or poor prognosis for return to function. A long-term follow-up was available for 41 (73.2%) cases out of the 56 cases that survived to discharge, with 25 (60.9%) cases out of the 41 cases surviving above 1 year after hospital discharge. Reasons for euthanasia at long-term follow-up were reported in 12 cases, which included unresolving septic synovitis (7 cases, 58.3%), unresolving colic signs (3 cases, 25%), and severe osteoarthritis due to septic synovitis (2 cases, 16.7%). A long-term return to function information was available for 15 (60%) cases out of the 25 cases that survived greater than 1 year. A total of 10 (66.7%) cases among those cases returned to their previous level of function, and 5 (33.3%) cases returned to a lower level.

The results of group 1 univariate logistic regression analysis and Fisher’s exact test are presented in Supplementary Table S1. For multiple logistic regression analysis, stepwise, backward, and Akaike information criterion (AIC) selections were used, which ended up with the same final models. The results of the multiple logistic regression analysis for group 1 are presented in Table 2. The steps for conducting multiple logistic regression analysis for group 1 are presented in Supplementary Table S2. Survival to hospital discharge was positively associated with the number of daily consecutive IVRLP (OR = 19.9; 95% CI: [1.49, 266.15]), intrasynovial antibiotics administered postoperatively (OR = 194.2; 95% CI: [2.03, ∞]), and the presence of intrasynovial fibrin (OR = 22.39; 95% CI: [0.64, 782]). Table 3 presents the number of cases for each group and the count of daily consecutive IVRLP treatments they received. The total number of IVRLP treatments during hospitalization was negatively associated with survival to hospital discharge (OR = 0.13; 95% CI: [0.02, 0.71]).

**Table 2:** Multiple logistic regression analysis for group 1 (septic synovitis cases) and group 2 (penetrating wound cases) variables included in the model and association for survival to hospital.

Variable	Category	OR	95% CI	*p*-value
Septic synovitis cases (group 1)				
Number of consecutive daily IVRLP	Daily IVRLP increasing number by one IVRLP without a 24-h interval between treatments	19.9	1.49, 266.15	0.02
Total number of IVRLPs	Number of IVRLPs increasing with each IVRLP administered	0.13	0.02, 0.71	0.02
Intrasynovial antibiotics postoperatively	Yes	194.2	2.03, ∞	0.02
	No	Reference		
Intrasynovial fibrin present	Yes	22.39	0.64, 782	0.09
	No	Reference		
Penetrating wound cases (group 2)				
Total number of IVRLPs	Number of IVRLPs increasing with each IVRLP administered	0.33	0.1, 1.1	0.07

*p ≤ 0.1.*

**Table 3:** The number of consecutive IVRLP treatment days and associated survival of cases to hospital discharge.

Consecutive IVRLP (days)	Survival (N)	Non-survival (N)	Total (N)
2	12	2	14
3	28	4	32
4	13	1	14
5	2	0	2
6	1	0	1

Group 2 lameness scores were recorded in 59 (59%) of 100 cases, and they subjectively improved by at least one (according to AAEP) lameness score within a median of 1 day after presentation (IQR: 1–3 days). Horses were hospitalized for a median of 8 days (IQR: 1–81). The short-term survival rate for group 2 was 99% (99 cases). The only case that did not survive was euthanized due to pedal osteolysis and joint collapse 7 days after a puncture wound to the distal interphalangeal joint. However, three cases from group 2 were discharged from the hospital, re-presented, and included in group 1 with established septic synovitis. Long term follow-up was available for 55 cases with 46 (83.6%) surviving more than a year after hospital discharge. The reasons for euthanasia during long-term follow-up were reported in five cases; these reasons included lameness or pain related to the original injury in two cases, pulmonary embolism in one case, clostridial disease in 1 case, and large colon volvulus in one case. Information on long-term return to function was available for 41 (89.1%) of 46 cases that survived longer than 1 year. Among the 41 cases, 31 (75.6%) cases returned to their previous level of function, while 10 (24.4%) cases returned to a lower level.

The results of univariate logistic regression analysis and Fisher’s exact test of group 2 are presented in Supplementary Table S1. For the multiple stepwise logistic regression analysis, stepwise, backward, and AIC selection methods were employed, all resulting in the same final models. The results of the multiple logistic regression analysis for group 2 are presented in Table 2. The steps for multiple logistic regression analysis for group 2 are reported in Supplementary Table S3. The total number of IVRLPs during hospitalization was negatively associated with survival to hospital discharge (OR = 0.33; 95% CI: [0.1, 1.1]).

The analysis of variables collected across both groups (*n* = 163) found that horses categorized in group 1 were less likely to survive to hospital discharge (*p* = 0.01; OR = 0.08; 95% CI: [0.01, 0.68]).

### 3.7 Re-presented cases

Four cases were presented once again to ISU LVMC after initial hospital discharge for SW or SS. Initially, three out of four cases were initially diagnosed as SW during their first presentation and then diagnosed as SS due to their second presentation. One case was presented for SS at two separate events.

The SS case involved a postinjection septic right tibiotarsal joint with a positive bacterial culture of *Staphylococcus epidermidis*. This case was initially treated locally with intrasynovial gentamicin 200 mg and gentamicin IVRLP every 24 h for 3 days followed by a 24-h interval without IVRLP and then one additional IVRLP. Systemically this horse was treated with SMZ/TMS. This horse was hospitalized for 5 days and then discharged from the hospital. After discharge, this case was presented again 7 days later for increased lameness and swelling of the right tibiotarsal joint. A computed tomography (CT) scan of the right tarsal region was performed, which showed erosion of the medial trochlea of the talus and cytology revealed SS. Arthroscopy was performed, following CT scan, with the removal of fibrin. This horse was treated with ceftiofur sodium both via IVRLP and systemically. The IVRLPs were administered every 24 h for 3 days. This horse was discharged from the hospital alive after 7 days.

For the three cases that were initially SW, Horse 1 was initially a left front digital flexor tendon sheath laceration treated with NTN lavage, systemic antibiotics (PPG, gentamicin) and gentamicin IVRLP. The IVRLP were performed every 24 h for 3 consecutive days with a 24-h interval without IVRLP followed by 2 consecutive days of IVRLP. Horse 1 was hospitalized for 12 days and then discharged. Horse 1 was presented once again 1 month after initial presentation with the same synovial structure affected. Synovial fluid culture was positive for *Staphylococcus* sp. and *Actinomyces* sp. Treatment was NTN lavage, placement of a Jackson-Pratt drain in surgery, systemic antibiotics (PPG, gentamicin), and IVRLP gentamicin. Horse 1 was hospitalized for 38 days before the second hospital discharge.

Horse 2 was initially presented with a laceration of the left hind dorsal fetlock and long digital extensor tendon. The case was treated with NTN lavage, intrasynovial gentamicin, IVRLP gentamicin, and systemic antibiotics (PPG, gentamicin). Systemic antibiotics were continued for 5 days followed by a change to SMZ/TMS. The IVRLP was performed every 24 h for 3 consecutive days, followed by a 24-h interval, without IVRLP followed by 3 consecutive days of IVRLP at 24-h intervals. Horse was hospitalized for 15 days and then discharged. Horse 2 was presented once again 1 week after hospital discharge with a septic left hind fetlock. Culture was positive for *E. coli*. Horse 2 was treated with fetlock arthroscopy, IVRLP meropenem for 4 consecutive days, and systemic antibiotics (enrofloxacin). The horse was hospitalized for 75 days, including hospitalization for breeding management. Horse 2 was confirmed to be 14 days in foal at hospital discharge.

Horse 3 initially presented with a laceration over the left plantar tibiotarsal and calcaneal bursa region. The horse was initially treated with arthroscopy, bursoscopy, IVRLP gentamicin, and systemic antibiotics (PPG, gentamicin). Three consecutive IVLRP every 24 h were performed. The horse was hospitalized for 7 days and then discharged from the hospital. The horse was presented once again 1 month later. At this time, calcaneal bursoscopy was performed with abscess drainage of a subcutaneous abscess proximal to the calcaneal bursa. The bursa and abscess were packed with absorbable antibiotic impregnated beads. The beads were impregnated with gentamicin. The horse was treated with a gentamicin IVRLP for 5 consecutive days and systemic antibiotics (PPG, gentamicin). The horse was hospitalized for 16 days and then discharged.

All horses survived to hospital discharge with three horses surviving more than a year after hospital discharge. The long-term information for these three horses was as follows: One horse survived more than a year, but no further information was provided, one horse was pasture sound, but did not return to athleticism, and one horse returned to its previous level of exercise.

## 4 Discussion

This retrospective study presents updated information on the short- and long-term survival of adult horses diagnosed with SS or SW treated with IVRLP at a single equine referral hospital in the Midwest United States from 2010 to 2020. Additionally, this study provides information regarding IVRLP variables associated with short-term survival in SS and SW cases. This study aimed to identify variables, if any, of IVRLP that were significantly associated with short- and long-term outcomes. The limited long-term follow-up prevented accurate variable analysis. For both SS and SW, an increasing number of IVRLPs was negatively associated with survival on multivariate analysis. Increasing total numbers of IVRLP often occur in cases that are not responding to therapy as quickly as expected. Poor response to treatment can be related to unresolved SS, persistent lameness, bacterial resistance, or development of other synovial pathology, all of which carry a poor prognosis for short-term survival (1, 13, 14, 16, 18, 19). Some of the cases in the current study identified these problems as part of the reason for euthanasia prior to discharge.

Interestingly, for SS cases an increasing number of daily consecutive IVRLP before an extended time break was positively associated with survival to discharge. In the current study, consecutive IVRLP were performed at consistent intervals. A break was considered a period when an IVRLP was not performed for at least twice the consistent interval (i.e., 48-h break when IVRLP had been performed every 24 h). The most frequent number of IVRLPs performed prior to a break for cases in this study was 3 (Table 3), followed by a time break, before a fourth IVRLP was performed. This result appears to stand in contrast to the previous finding that increasing total numbers of IVRLP was negatively associated with survival. It is possible that increasing numbers of IVRLP prior to an extended break reduces bacterial escape and produces a better bactericidal effect (15). Gentamicin in IVRLP at amounts (500 mg) lower than the median amount in this study (1,000 mg) was shown to achieve concentrations 10 times MIC (10). It would be reasonable to assume that IVRLP in the current study would achieve similar or better synovial concentrations. These concentrations are above the resistance threshold and achieve maximal antimicrobial effect. Without an ideal number of IVRLPs identified, it would be reasonable to follow the clinical course of each patient in determining how many consecutive IVRLPs would be most appropriate. None of the other examined IVRLP variables had a statistically significant effect on short-term outcomes for SS and SW cases.

Gentamicin was the primary antimicrobial in IVRLP for 93.9% (153/163) of cases in this study. The use of gentamicin as the primary antibiotic in IVRLP has been studied for penetration and concentration within septic synovial structures for resolution of SS or SW (3, 4, 10, 20). The use of gentamicin for IVRLP at this referral hospital as empirical IVRLP treatment has been elected based on these reports and synovial fluid bacterial cultures from the hospital’s microbiology laboratory (3, 4, 10, 20). Gentamicin as a primary antimicrobial in horses is effective in 70–83.9% of bacterial isolates from musculoskeletal infections in the horse, and 40–100% effective on non-*Streptococcus* isolates from synovial samples in adult horses (21–23). Gentamicin has reportedly increased irritation to the vasculature, however, this was not evaluated in this study, and was not noted in the records for the cases as an observation (24). There were only 10 cases where gentamicin was not used or switched to another antibiotic, preventing comparison of outcomes between antimicrobials. Reasons for antimicrobial switching were not recorded in the medical records but likely were due to clinician preference or bacterial culture sensitivity results. The outcomes of empirical gentamicin use in IVRLP for SS and SW cases in a large retrospective study have not been reported to date. The results of this study suggest that gentamicin IVRLP can be a safe and potentially effective empirical IVRLP antimicrobial in the treatment of synovial wounds and infections for horses in the Midwest United States. Use of gentamicin as a systemic antimicrobial in SS and SW cases is common (87.7% in the current study). Incorporation of gentamicin into the IVRLP reduces the need for additional aminoglycosides and reduces the number of concurrent antimicrobials used on a patient. It is recommended that veterinarians contact their local laboratory to determine which antimicrobial may be most effective in empirical treatment in their area.

The use of intrasynovial antimicrobials was a non-IVRLP variable that was found to be positively associated with short-term survival in SS cases. Intrasynovial antimicrobial administration has been suggested as an adjunctive therapy that may improve outcomes in synovial sepsis cases in early literature (25). However, to the authors’ knowledge there are no reports that experimentally or clinically evaluate the combined administration of IVRLP and intrasynovial antibiotics. The authors presume that a combination therapy of IVRLP and intrasynovial antibiotics would increase the intrasynovial antibiotic concentration, increasing bacterial killing. Future studies would be beneficial in identifying these concentrations as it was found that aminoglycosides have a chondrotoxic effect *in vitro*, suggesting that intrasynovial antimicrobials have a detrimental effect on the synovial cartilage (26). Determining if the concentrations resulting from antibiotic IVRLP and intrasynovial administration would lead to these chondrotoxic effects would help determine if the doses used clinically are effective or may lead to potential chondrotoxicity.

This study found that short-term survival was 88.9 and 99% for SS and SW cases, respectively. This study used strictly defined inclusion criteria in a previous study to separate established SS from SW cases (1). These criteria have not been uniformly applied in previous studies, making direct comparisons of survival rates difficult. The previous studies examining SS reported short-time survival rates (71.9–87%) consistent with the SS survival rates in the current study (1, 2). For the SW cases the short-term survival rate was higher than previous reports (87–94%) (1, 2, 18). The long-term follow up for both cohorts in the present study was limited, but survival more than a year was 60.9% for SS and 83.6% for SW. SS long-term survival rates in the literature are 53.4–90 and 87–94% for SW, consistent with the present study despite limited data (1–3, 16, 18, 19, 27). The increase in SW survival may reflect the contemporary context of this study compared to the reference literature, which was published in the mid-2000s, and could be a result of client education, earlier treatment, or utilizing contemporary IVRLP techniques (1–11). However, reports may not have clear separation between SS and SW, making comparison among survival rates challenging (2, 18, 19). The findings reported in this study suggest that short-term survival of equine SS and SW cases receiving IVRLP is good to excellent while long-term survival may be guarded to good (1–3).

The return to function after SS or SW is a primary concern for owners and veterinarians when treating these cases. In this report, horses treated with IVRLP survived more than a year and returned to their previous level of exercise were 75.6% (46/55). This is similar to an IVRLP study with identical inclusion criteria (72.7%) and higher than another IVRLP report that did not differentiate between SS and SW (61%) (1, 2). The return to the same level of performance for SS horses available for long-term follow-up was 60% (15/25), and 75.6% (31/41) for SW horses. Regardless of IVRLP use, a return to function has been reported between 36 and 81% for septic synovitis in equine, (1, 3, 16, 18, 19, 27). The variations in survival and return to previous level of exercise in the current study may be related to case inclusion based on age, criterion for SS inclusion, or potential improvements in client education, clinical care, or IVRLP technique compared to other clinical reports. Overall, the short-term survival for SW appears excellent with a good prognosis for return to previous athletic function (1, 2). Further prospective studies evaluating IVRLP therapy using strict inclusion criterion between SW and SS would be beneficial in assessing survival and return to function for these cases.

Primary limitations of this study include its retrospective design and limited long-term follow-up response. Cases with incomplete information or values may influence the final statistical significance of certain variables. Other cases may have not been included due to IVRLP use not being recorded correctly in the medical record. Cases did not denote involvement of different structures like tendons and ligaments, or concurrent pathology, which has been shown to influence short- and long-term outcomes (17–18,20). A control population was not retrospectively identified in this study and would have helped strengthen the statistical outcomes identified. Often, IVRLP is considered the standard of care for SS and SW; treating veterinarians and identifying matched control cases that had similar lesions and did not receive IVRLP would be challenging due to the perceived lack of standard of veterinary care. It would be interesting to perform a prospective study comparing horses treated with IVRLP versus intrasynovial antimicrobial injection. Additionally, in our current study, 153/163 (93.8%) cases had either endoscopic or needle-through-needle lavage performed. This makes distinguishing effects of IVRLP administration from surgical lavage extremely difficult. Creating a case–control study to evaluate the impact of IVRLP specifically on outcomes of SS or SW may be a valuable future investigation. The limited follow-up response for cases in this study impeded the ability to draw significant conclusions about different IVRLP variables affecting long-term outcomes. Cases identified in the study may have been sold, client contact information may have changed, or clients may no longer have an affiliation with the referral institution during that period. These factors and more all contribute to the difficulty in collecting large numbers of reliable long-term follow-up details, reducing the statistical significance of variables on outcomes.

Overall, this study provided an update on the clinical outcomes of equine SS and SW cases receiving IVRLP as a part of their treatment protocol from 2010 to 2020 and identifies variables associated with IVRLP for short-term outcome. The study outcomes were consistent with the current literature. The results of this study suggest that performing more consecutive IVRLP prior to a break may be positively associated with short-term outcome rates for SS cases. However, future prospective investigations would be helpful. Future studies with a control group that does not receive IVRLP or a comparison group only receiving intrasynovial antibiotics would be beneficial. Additionally, this study suggests that empirical IVRLP treatment with gentamicin may be a potential option for SS or SW located within the midwestern region studied in this paper.

## Data Availability

The raw data supporting the conclusions of this article will be made available by the authors, without undue reservation.
