# Supplementary material for: Clinical use of antimicrobial regional limb perfusion in adult horses diagnosed with synovial sepsis or penetrating synovial wounds at a single equine referral hospital in the Midwest United States—163 cases (2010–2020)
Source: Front Vet Sci. 2025 Mar 26;12:1504486. doi: 10.3389/fvets.2025.1504486 (PMC11979138; doi:10.3389/fvets.2025.1504486)
Supplement: Supplementary file 1 [file Data_Sheet_1.docx]

**Supplemental Document 1:** Telephone Questionnaire for Long-Term Follow Up with Clients

Question 1: Is the “patient” still alive?

a. If no longer living, when did they die?

b. What was the cause of death?

Question 2: Did the “patient” demonstrate lameness after discharge?

1. Was the lameness on the same limb as the presenting complaint?
2. Was the lameness related to the initial injury?

Question 3: Was the “patient” treated for the lameness or joint infection by a veterinarian?

a. If yes, was there a culture and sensitivity performed?

Question 4: Was the “patient” able to return to their previous level of exercise or use after discharge?
