# Supplementary material for: Clinical use of antimicrobial regional limb perfusion in adult horses diagnosed with synovial sepsis or penetrating synovial wounds at a single equine referral hospital in the Midwest United States—163 cases (2010–2020)
Source: Front Vet Sci. 2025 Mar 26;12:1504486. doi: 10.3389/fvets.2025.1504486 (PMC11979138; doi:10.3389/fvets.2025.1504486)
Supplement: Supplementary file 2 [file Table_1.docx]

**Supplemental Table 1:** Individual variables tested in each group by logistic regression analysis and Fisher’s exact test. P-value <0.05 was considered significant. Odds ratios and 95% confidence intervals are included if they were reported.

| **Variable of Interest** | **Group 1 (SS)** | | | **Group 2 (SW)** | | |
| --- | --- | --- | --- | --- | --- | --- |
|  | p-value | Odds Ratio | 95% Confidence Interval | p-value | Odds Ratio | 95% Confidence Interval |
| Sex | 0.60 | n/r | n/r | 0.48 | n/r | n/r |
| Synovial Structure Involved | 0.10 | 1.18 | [0.973 - 1.478] | 0.18 | 1.46 | [0.824 - Undefined] |
| Synovial Lavage Type | 1.00 | n/r | n/r | 0.87 | n/r | n/r |
| Hospitalization Duration | 0.59 | 0.99 | [0.947 - 1.056] | 0.16 | 0.95 | [0.889 - 1.030] |
| IVRLP Administration Site | 0.12 | n/r | n/r | 0.35 | n/r | n/r |
| Initial IVRLP GA vs Standing | 0.34 | 1.09 | [0.191 - 6.249] | 0.70 | 0.99 | [0.958 - 1.014] |
| IVRLP Antibiotics | 0.33 | n/r | n/r | 0.96 | n/r | n/r |
| Total IVRLP Volume | 1.00 | 1.01 | [0.941 - 1.096] | 1.00 | 0.99 | [0.883 - 1.191] |
| Use of Local Anesthetic in IVRLP | 0.26 | 0.53 | [0.095 - 2.988] | 0.71 | 0.99 | [0.959 - 1.014] |
| IVRLP Local Anesthetic Type | 0.72 | n/r | n/r | 0.69 | n/r | n/r |
| Use of Perineural Block | 0.20 | 0.11 | [0.006 - 1.977] | 0.99 | 1.01 | [0.990 - 1.030] |
| Number of IVRLP Before Break | 0.56 | 1.57 | [0.531 - 5.767] | 1.00 | 0.84 | [0.096 - 18.106] |
| Total Number of IVRLP* | 0.01 | 0.47 | [0.216 - 0.865] | 0.08 | 0.37 | [0.082 - 1.135] |
| Time Interval Between IVRLP | 1.00 | 1.04 | [0.854 - 1.609] | 1.00 | 1.03 | [0.950 - 1.402] |
| Intraarticular Antibiotics | 0.08 | 0.25 | [0.050 - 1.256] | 0.18 | 0.94 | [0.844 - 1.056] |
| Organism Type Seen on Cytology | 0.39 | n/r | n/r | 0.86 | n/r | n/r |
| Fibrin in Synovial Space | 0.11 | 4.84 | [0.546 - 42.872] | 0.83 | 1.01 | [0.988 - 1.037] |
| Positive Bacterial Culture | 0.31 | 0.89 | [0.810 - 0.986] | n/r | n/r | n/r |

(*) = Variable found to be significant on simple logistic regression analysis for Group 1.
