# Supplementary material for: Clinical use of antimicrobial regional limb perfusion in adult horses diagnosed with synovial sepsis or penetrating synovial wounds at a single equine referral hospital in the Midwest United States—163 cases (2010–2020)
Source: Front Vet Sci. 2025 Mar 26;12:1504486. doi: 10.3389/fvets.2025.1504486 (PMC11979138; doi:10.3389/fvets.2025.1504486)
Supplement: Supplementary file 3 [file Table_2.docx]

**Supplemental Table 2:** Multiple logistic regression analysis for Group 1 (septic synovitis cases) for survival to hospital discharge non-selected and selected variables for the final model.

| **Summary Of Backward Elimination (Non-Selected Variables)** | | | | | | | |
| --- | --- | --- | --- | --- | --- | --- | --- |
| **Step** | **Variable Removed** | | **DF** | | **Number In** | **Wald Chi-Square** | **Pr>ChiSq** |
| 1 | Breed | | 8 | | 15 | 0.16 | 1.0 |
| 2 | Time Interval Between IVRLP | | 1 | | 14 | <0.01 | 0.98 |
| 3 | Synovial Lavage (Endoscopy; Needle) | | 3 | | 13 | 0.1679 | 0.98 |
| 4 | IVRLP Administration Vein | | 4 | | 12 | 0.21 | 0.99 |
| 5 | Synovial Structure Type Involved | | 1 | | 11 | 0.01 | 0.91 |
| 6 | Organisms Seen on Cytology | | 2 | | 10 | 0.2 | 0.9 |
| 7 | IVRLP Antibiotic Type | | 1 | | 9 | 0.08 | 0.78 |
| 8 | Use of IV Anesthetic in IVRLP solution | | 1 | | 8 | 0.02 | 0.89 |
| 9 | Use of Perineural Anesthesia | | 1 | | 7 | 0.09 | 0.77 |
| 10 | Sex (male, female, gelding) | | 2 | | 6 | 1.35 | 0.51 |
| 11 | Hospitalization Time (days) | | 1 | | 5 | 0.99 | 0.32 |
| 12 | Initial IVRLP Performed under General Anesthesia or Standing Sedation | | 1 | | 4 | 0.32 | 0.57 |
|  | | | | | | | |
| **Type 3 Analysis of Effects (Selected Variables)** | | | | | | | |
| **Effect** | | **DF** | | **Wald Chi-Square** | | | **Pr>ChiSq** |
| Number of Consecutive Daily IVRLP | | 1 | | 5.11 | | | 0.02 |
| Total Number of IVRLP | | 1 | | 5.5 | | | 0.02 |
| Intrasynovial Antibiotics Postoperatively | | 1 | | 5.12 | | | 0.02 |
| Intrasynovial Fibrin Present | | 1 | | 2.94 | | | 0.09 |
