# Supplementary material for: Clinical use of antimicrobial regional limb perfusion in adult horses diagnosed with synovial sepsis or penetrating synovial wounds at a single equine referral hospital in the Midwest United States—163 cases (2010–2020)
Source: Front Vet Sci. 2025 Mar 26;12:1504486. doi: 10.3389/fvets.2025.1504486 (PMC11979138; doi:10.3389/fvets.2025.1504486)
Supplement: Supplementary file 4 [file Table_3.docx]

**Supplemental Table 3:** Multiple logistic regression analysis for Group 2 (penetrating wound cases) for survival to hospital discharge non-selected and selected variables for the final model.

| **Summary Of Backward Elimination (Non-Selected Variables)** | | | | | | | |
| --- | --- | --- | --- | --- | --- | --- | --- |
| **Step** | **Variable Removed** | | **DF** | | **Number In** | **Wald Chi-Square** | **Pr>ChiSq** |
| 1 | Breed | | 10 | | 15 | <0.01 | 1.0 |
| 2 | IVRLP Administration Vein | | 5 | | 14 | 0.02 | 1.0 |
| 3 | Synovial Lavage (Endoscopy; Needle) | | 3 | | 13 | <0.01 | 1.0 |
| 4 | Sex (male, female, gelding) | | 2 | | 12 | <0.01 | 0.99 |
| 5 | Time Interval Between IVRLP | | 1 | | 11 | <0.01 | 0.99 |
| 6 | Use of IV Anesthetic in IVRLP solution | | 1 | | 10 | <0.01 | 0.99 |
| 7 | Initial IVRLP Performed under General Anesthesia or Standing Sedation | | 1 | | 9 | <0.01 | 0.99 |
| 8 | Organisms Seen on Cytology | | 1 | | 8 | <0.01 | 0.99 |
| 9 | IVRLP Antibiotic Type | | 2 | | 7 | 0.06 | 0.97 |
| 10 | Intrasynovial Fibrin Present | | 1 | | 6 | <0.01 | 0.968 |
| 11 | Number of Consecutive Daily IVRLP | | 1 | | 5 | <0.01 | 0.97 |
| 12 | Use of Perineural Anesthesia | | 1 | | 4 | <0.01 | 0.95 |
| 13 | Synovial Structure Type Involved | | 1 | | 3 | 0.13 | 0.72 |
| 14 | Hospitalization Time (days) | | 1 | | 2 | 0.74 | 0.39 |
| 15 | Intrasynovial Antibiotics Postoperatively | | 1 | | 1 | 0.02 | 0.89 |
|  | | | | | | | |
| **Type 3 Analysis of Effects (Selected Variables)** | | | | | | | |
| **Effect** | | **DF** | | **Wald Chi-Square** | | | **Pr>ChiSq** |
| Total Number of IVRLP | | 1 | | 3.2 | | | 0.07 |
